# *Pichia pastoris*-expressed dengue 3 envelope-based virus-like particles elicit predominantly domain III-focused high titer neutralizing antibodies

**DOI:** 10.3389/fmicb.2015.01005

**Published:** 2015-09-23

**Authors:** Lav Tripathi, Shailendra Mani, Rajendra Raut, Ankur Poddar, Poornima Tyagi, Upasana Arora, Aravinda de Silva, Sathyamangalam Swaminathan, Navin Khanna

**Affiliations:** ^1^Recombinant Gene Products Group, International Centre for Genetic Engineering and Biotechnology, New DelhiIndia; ^2^Department of Microbiology and Immunology, University of North Carolina School of Medicine, Chapel Hill, NCUSA; ^3^Department of Biological Sciences, Birla Institute of Technology and Sciences, HyderabadIndia; ^4^Translational Health Science and Technology Institute, NCR Biotech Science Cluster, FaridabadIndia; ^5^Department of Pediatrics, Emory University School of Medicine, Atlanta, GAUSA

**Keywords:** dengue, dengue envelope, dengue vaccine, virus-like particle (VLP), neutralizing antibody, antibody-dependent enhancement (ADE), *Pichia pastoris*

## Abstract

Dengue poses a serious public health risk to nearly half the global population. It causes ~400 million infections annually and is considered to be one of the fastest spreading vector-borne diseases. Four distinct serotypes of dengue viruses (DENV-1, -2, -3, and -4) cause dengue disease, which may be either mild or extremely severe. Antibody-dependent enhancement (ADE), by pre-existing cross-reactive antibodies, is considered to be the major mechanism underlying severe disease. This mandates that a preventive vaccine must confer simultaneous and durable immunity to each of the four prevalent DENV serotypes. Recently, we used *Pichia pastoris*, to express recombinant DENV-2 E ectodomain, and found that it assembled into virus-like particles (VLPs), in the absence of prM, implicated in the elicitation of ADE-mediating antibodies. These VLPs elicited predominantly type-specific neutralizing antibodies that conferred significant protection against lethal DENV-2 challenge, in a mouse model. The current work is an extension of this approach to develop prM-lacking DENV-3 E VLPs. Our data reveal that *P. pastoris*-produced DENV-3 E VLPs not only preserve the antigenic integrity of the major neutralizing epitopes, but also elicit potent DENV-3 virus-neutralizing antibodies. Further, these neutralizing antibodies appear to be exclusively directed toward domain III of the DENV-3 E VLPs. Significantly, they also lack discernible ADE potential toward heterotypic DENVs. Taken together with the high productivity of the *P. pastoris* expression system, this approach could potentially pave the way toward developing a DENV E-based, inexpensive, safe, and efficacious tetravalent sub-unit vaccine, for use in resource-poor dengue endemic countries.

## Introduction

Dengue is currently regarded by the World Health Organization (WHO) as one of the fastest spreading vector-borne diseases (World Health Organization [WHO], 2012). It is prevalent in >100 countries around the world, placing >2.5 billion people at risk. Recent estimates indicate that there may be as many as 400 million infections annually (Bhatt et al., 2013). The disease, which is caused by four closely related, but antigenically distinct, serotypes of dengue viruses (DENV-1, -2, -3, and -4), is spread to humans by mosquitoes of the genus *Aedes*. Dengue infections may be inapparent, mild [dengue fever, (DF)] or severe [dengue hemorrhagic fever (DHF), and dengue shock syndrome (DSS], with potentially fatal outcomes in the absence of medical care (Gubler et al., 2007; Swaminathan and Khanna, 2009; World Health Organization [WHO], 2015). The severe disease is believed to be the result of pre-existing cross-reactive antibodies which apparently facilitate uptake of heterotypic DENVs into monocytes and macrophages through FcγR pathway, a phenomenon termed as antibody-dependent enhancement (ADE; Halstead, 2003; Gubler et al., 2007). This situation warrants a safe dengue vaccine to confer simultaneous and durable immunity to each of the four prevalent DENV serotypes. This, together with lack of clear definition of correlates of protection and a reliable animal model have made dengue vaccine an elusive goal. The challenges inherent in dengue vaccine development are underscored by the lack of clarity on efficacy despite the conclusion of three efficacy trials to date, of a live attenuated vaccine (LAV) candidate for dengue (Sabchareon et al., 2012; Capeding et al., 2014; Villar et al., 2015). This, and the uncertainty associated with LAVs stemming from the potential for imbalances in immunity due to immune interference (Edelman, 2011; Thomas, 2011; Swaminathan et al., 2013) have re-focused attention on dengue vaccine initiatives based on non-replicating sub-unit vaccine candidates (Schmitz et al., 2011).

In this context, it is noteworthy that several viral structural proteins possess an intrinsic potential to self-assemble into virus-like particles (VLPs). It is well-documented that structural proteins of many viruses, produced recombinantly in heterologous hosts, form VLPs (Chackerian, 2007; Wang et al., 2009; Liu et al., 2010; Kuwahara and Konishi, 2010; Tang et al., 2012). The unique feature of the VLP is that it displays repetitive epitope arrays on its surface, while at the same time lacking infectious viral genomic material. Thus, the VLP is not only highly immunogenic, capable of eliciting a robust immune response, but also safe, as it is non-infectious (Bachmann and Jennings, 2010; Yildiz et al., 2011). This makes the VLP an ideal vaccine platform, as evidenced by the success of VLP vaccines for hepatitis B and human papilloma virus infections (Chackerian, 2007).

In the context of DENV, the major structural antigen from the perspective of a vaccine candidate is the envelope (E) glycoprotein. This is a large, ~500 amino acid (*aa*) residue protein, whose C-terminal ~100 *aa* residues are embedded in the host-membrane on the surface of the mature virion (Lindenbach et al., 2007). The N-terminal 80% (known as the ectodomain) is organized into distinct sub-domains, envelope domain I (EDI), EDII, and EDIII, stabilized by six S–S linkages (Modis et al., 2003). Of these, EDIII which is implicated in host receptor recognition, also contains multiple potent and type-specific neutralizing epitopes (Gromowski and Barrett, 2007; Shrestha et al., 2010). The minor structural protein, prM, which has a role in virus maturation (Lindenbach et al., 2007), is implicated in the induction of antibodies that can mediate ADE (Dejnirattisai et al., 2010; Rodenhuis-Zybert et al., 2010). Reports in the literature have led to the conclusion that co-expression of both these DENV structural proteins in heterologous host systems is required to produce VLPs (Wang et al., 2009; Liu et al., 2010; Kuwahara and Konishi, 2010; Tang et al., 2012).

Recently, using the methylotrophic yeast *Pichia pastoris* as the expression host, we showed that the DENV-2 E ectodomain, assembled into highly immunogenic VLPs. It is significant that these VLPs were formed in the absence of prM and induced potent DENV-2 virus-neutralizing antibodies which conferred significant protection against lethal challenge in a mouse model (Mani et al., 2013). The lack of prM eliminates the associated risk of ADE from these VLPs and is clearly a safety advantage. From the perspective of inexpensive production of recombinant sub-unit vaccines, the availability of a very strong methanol-inducible alcohol oxidase 1 (*AOX1*) promoter, the propensity of *P. pastoris* to grow to high cell densities in simple inexpensive media, its capacity for high productivity and ability to execute post-translational modifications, make this yeast a robust and desirable heterologous expression system (Macauley-Patrick et al., 2005). This opens up the feasibility of developing an inexpensive, safe and efficacious tetravalent sub-unit vaccine based on *P. pastoris*-produced E VLPs of all four DENV serotypes. In a step in this direction, we demonstrate in the current work that it is possible to adopt a similar approach to create DENV-3 E (ectodomain) VLPs (hereafter referred to as DENV-3 E VLPs for simplicity). We further show that these VLPs elicit potent virus-neutralizing antibodies. Interestingly, the neutralizing antibodies elicited by the DENV-3 E VLPs appear to be predominantly homotypic, that is, specific to DENV-3. Antibody depletion experiments suggest that the neutralizing antibodies are exclusively directed toward the EDIII of the DENV-3 E VLPs. Importantly, the antibodies elicited by the DENV-3 E VLPs did not manifest significant ADE.

## Materials and Methods

### Ethics Statement

Animal experiments strictly adhered to the guidelines set out by the Committee for the Purpose of Control and Supervision of Experiments on Animals (CPCSEA), Government of India. Animal experimental protocols were approved by the Institutional Animal Ethics Committees of International Centre for Genetic Engineering & Biotechnology, New Delhi, and Syngene International Limited, Bangalore (IAEC No. Syngene/IAEC/520/06-2014).

### *DENV-3 E* Gene, Plasmid, Cell Hosts, Viruses and Other Reagents

The *DENV-3 E* gene (~1.4 Kb, GenBank accession no: JX292266) was custom-synthesized by BioBasic Inc., Canada. This synthetic gene was codon-optimized for expression in *P. pastoris*. *P. pastoris* strain KM71H and plasmid pPICZ-A were from Invitrogen Life Technologies (Carlsbad, CA, USA). pPICZ-A is an integrative plasmid which provides the methanol-inducible *AOX1* promoter for transgene expression and the zeocin resistance marker for selection. Vero and BHK 21 cell lines were from American Type Culture Collection (ATCC), Virginia, USA. The WHO reference DENV-1, DENV-2, DENV-3, and DENV-4 viruses were the same as reported earlier (Kraus et al., 2007). Ni^2+^-NTA His-Sorb plates and Ni^2+^-NTA Super-flow resin were obtained from Qiagen (Hilden, Germany). DENV-2 EDIII-specific mAb 24A12 (Mani et al., 2013) and prM-specific 2H2 mAb (Martin et al., 2006) have been reported earlier. 4G2 mAb was from ATCC. All other type-specific and cross-reactive human and murine mAbs have been described before (Henchal et al., 1982; Brien et al., 2010; Shrestha et al., 2010; Sukupolvi-Petty et al., 2010, 2013; Wahala et al., 2010; De Alwis et al., 2011; Smith et al., 2012, 2013). Secondary antibody conjugates for ELISA [anti-mouse IgG antibody-horseradish peroxidase (HRPO)] and indirect immunofluorescence assay (IFA) [IgG-fluorescene isothiocyanate (FITC) conjugates] were from Calbiochem, La Jolla, CA, USA. The HRPO substrate 3, 3′, 5, 5′-Tetramethylbenzidine (TMB), Concanavalin A (Con A)-HRPO conjugate and acid-washed glass beads (425–600 microns) were purchased from Sigma–Aldrich, St. Louis, MO, USA. Uranyl acetate was from TAAB Laboratories Equipment Ltd (UK).

### Expression and Purification of Recombinant DENV-3 E

The *DENV-3 E* gene was integrated into the genome of *P. pastoris* (strain KM71H) under the control of the *AOX1* promoter as done earlier for *DENV-2 E* gene. Expression was induced using methanol and the recombinant protein purified under denaturing conditions, using Ni^2+^ affinity chromatography, essentially as described before (Mani et al., 2013). The purified protein was characterized by SDS-PAGE, Western blot analysis and His Sorb ELISA (using mAb 24A12), protein blotting (with Con A-HRPO) to assess glycosylation status, and N-terminal sequence analysis, as reported recently (Mani et al., 2013).

Antigenic integrity of epitopes on the DENV-3 E protein was assessed using indirect ELISA. These ELISAs were done using purified *P. pastoris*-produced DENV-3 E as the coating antigen and a battery of previously reported type-specific and cross-reactive human and murine mAbs (Henchal et al., 1982; Brien et al., 2010; Shrestha et al., 2010; Sukupolvi-Petty et al., 2010, 2013; Wahala et al., 2010; De Alwis et al., 2011; Smith et al., 2012, 2013).

To examine VLP formation, purified DENV-3 E protein was examined by electron microscopy as described (Arora et al., 2012). Particle size and distribution of the VLPs (at 300 μg/ml in 20 mM Tris-HCl, pH8.5, containing 50 mM NaCl) were analyzed by Dynamic Light Scattering (DLS), using Malvern Zetasizer Nano Z.

### Mouse Immunization and Seroanalysis

Groups (*n* = 6) of ~6-week old BALB/c mice were immunized intra-peritoneally with 20 μg purified recombinant DENV-3 E antigen coated on alum, on days 0, 30, and 90, and sera collected ~7 days after the first and second boosts, as reported (Mani et al., 2013).

Antibodies induced by DENV-3 E VLPs were analyzed by indirect ELISAs and indirect IFA, essentially as described recently (Mani et al., 2013). Homotypic (specific to DENV-3) and heterotypic (specific to DENV-1, -2, and -4) neutralizing antibody titers were determined using a Fluorescence Activated Cell Sorting (FACS)-based assay (Kraus et al., 2007), as done earlier for anti-DENV-2 E VLP antiserum (Mani et al., 2013).

In some experiments the immune serum was depleted of antibodies specific to EDIII of DENV-3 E protein (EDIII-3), before being used in the FACS-based neutralization assay. Depletion was accomplished by pre-incubating the immune serum with amylose bound to maltose-binding protein (MBP) fused in-frame to EDIII-3. Depleted serum was then incubated with DENV-3 followed by infection of Vero cells and FACS analysis. Control sample was depleted using amylose-MBP instead of the fusion protein.

Fluorescence Activated Cell-based Neutralization Titer (FNT_50_) of the immune serum was defined as serum dilution resulting in a 50% reduction in the number of DENV-infected cells (with reference to DENV infection in the absence of immune serum).

Homotypic and heterotypic ADE was evaluated using a similar FACS-based assay, with the exception that FcγR-bearing K562 cells were used instead of Vero cells. The magnitude of ADE was expressed as fold-enhancement (percentage of K562 cells infected in the presence of antiserum divided by the corresponding percentage in the absence of antiserum).

### Statistical Analysis

Statistical significance of the difference between data sets was determined by two-tailed Student *t*-test, using GraphPad Prism software for Windows. Probability (*p*) levels less than 0.05 were considered as significant.

## Results

### Yeast-Expressed DENV-3 E Antigen Assembles into VLPs

We created a synthetic *DENV-3 E* gene and cloned it into the expression vector pPICZ-A under the control of the *AOX1* promoter, incorporated it into the genome of the *P. pastoris* host strain KM71H, and verified its expression after methanol induction using mAb 24A12 (Supplementary Figures S1 and S2), as reported earlier (Mani et al., 2013). As seen for DENV-2 E, the DENV-3E protein was also associated with the membrane fraction in the induced cell lysate. It was, therefore, purified under denaturing conditions (Supplementary Figure S3) from the membrane-enriched fraction using the Ni^2+^-NTA affinity chromatography method designed earlier for DENV-2 E protein purification (Mani et al., 2013). Further, as observed in the case of DENV-2 E protein, the DENV-3 E protein was also processed appropriately in *P. pastoris*, based on N-terminal sequencing and glycosylation studies. Yield of the purified recombinant DENV-3 E protein was ~15 mg/L induced culture.

**FIGURE 1:**
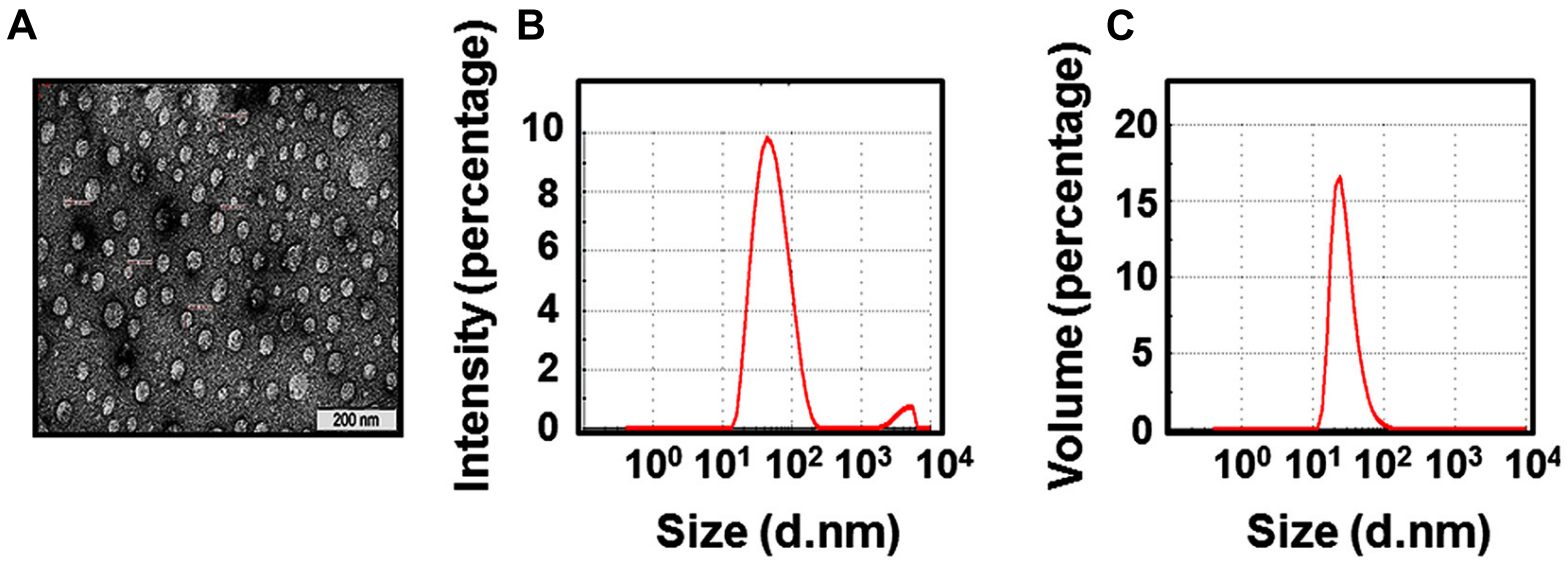
**Detection of virus-like particles (VLPs) in the purified DENV-3 E protein preparation. (A)** EM analysis of freshly purified DENV-3 E antigen following negative staining with 1% uranyl acetate. **(B)** Dynamic Light Scattering (DLS) analysis of particle size distribution by intensity. **(C)** DLS analysis of particle size distribution by volume.

We had observed earlier that the *P. pastoris*-expressed DENV-2 E protein is able to assemble into VLPs in the absence of prM (Mani et al., 2013). We therefore expected that the DENV-3 E protein produced using this yeast may also manifest this attribute. To verify this, we analyzed the purified recombinant DENV-3 E protein preparation by electron microscopy after negative staining with uranyl acetate, as shown in **Figure 1A**. Consistent with expectation, the ectodomain of DENV 3 E protein formed discrete VLPs, in the absence of prM. Given that purification was carried out in the presence of urea, VLP formation presumably occurred upon gradual urea removal during dialysis. The observation that *P. pastoris*-expressed recombinant DENV-3 E protein ectodomain assembles into VLPs is consistent with the behavior of its DENV-2 E counterpart, reported earlier (Mani et al., 2013). The EM data reveal that the DENV-3 VLPs ranged in size from 25 to 50 nm. Further, EM analysis revealed the VLPs to be intact after 2 weeks incubation at 37°C (*data not shown*). We also analyzed particle size in purified DENV3 E VLP preparation by laser-based DLS. This technique which monitors Brownian movement of the VLPs in native solution can yield information pertaining to average size and frequency distribution of particles. As shown in **Figure 1**, DLS data represented on the basis of either intensity (**Figure 1B**) or volume (**Figure 1C**), resulted in quite similar particle size distribution profiles with average VLP diameter of ~47 nm, which was in fair agreement with EM data.

### Overall Domain Architecture and Epitope Integrity of DENV-3 E are Preserved in the VLPs

We carried out an extensive immunological characterization of the yeast-expressed DENV-3 E VLPs using a panel of type-specific and cross-reactive murine and human mAbs obtained using E, EDI/II, EDIII and fusion loop antigens. For this, we employed an indirect ELISA using purified DENV-3 E VLPs as the coating antigen, followed by detection of DENV-3 E VLP-bound mAbs using cognate secondary Ab-HRPO enzyme conjugate (**Table 1**). For a comparison, we performed this ELISA in parallel using our *P. pastoris*-produced DENV-2 E VLPs, reported earlier (Mani et al., 2013). DENV-3 mAbs E3, E4, and E12, all specific to EDI/II region of the DENV-3 E antigen (Brien et al., 2010) bound efficiently to DENV-3 E VLPs (but not to DENV-2 E VLPs), suggesting that the EI/II region of the E antigen retains its native antigenic structure in the DENV-3 E VLPs. This is further supported by the observation that the DENV-3 E VLPs are also recognized by EI/II epitope-specific cross-reactive mAbs such as E17 (Brien et al., 2010) and h-23.13 (De Alwis et al., 2011), with the former binding more efficiently. In addition, the DENV-3 E VLPs also were recognized by human mAbs h-1N5 and h-1M7 (Smith et al., 2013), and the murine mAb 4G2 (Henchal et al., 1982). These mAbs are specific to the fusion loop of the DENV E protein.

**Table 1:** Analysis of antigenic integrity of DENV-3 and DENV-2 E VLPs*^a^.*

Type-specificmurine mAbs			
**mAb^b^**	**Epitope specificity**	**Absorbance, 450 nm**	
		**DENV-3 E**	**DENV-2 E**
DENV1 E24	EDIII	0.03	0.02
DENV1 E29	EDIII	0.09	0.03
DENV1 E37	EDIII	0.03	0.02
DENV1 E103	EDIII, LR	0.06	0.06
DENV-2 3H5	EDIII, LR	0.06	3.46
DENV-2 106	EDIII, LR	0.03	2.50
DENV-2 70	EDIII AS, LR	0.03	0.92
DENV-2 104	EDIII, C-C’ loop	0.10	3.45
DENV-3 E3	EDI/II	2.90	0.02
DENV-3 E4	EDI/II	3.18	0.04
DENV-3 E12	EDI/II	3.24	0.07
DENV-3 E1	EDIII	3.43	0.03
DENV-3 8A1	EDIII, LR	1.35	0.02
DENV-4 E88	EDIII, LR	0.03	0.07
DENV-4 E29	EDIII, F/G strand	0.03	0.04
DENV-4 E2	E	0.03	0.03
DENV-4 E42	E	0.02	0.02
DENV-4 E43	E	0.02	0.02

**Cross-reactive murine and human mAbs**			
**mAb**	**Epitope specificity**	**Absorbance, 450 nm**	
		**DENV-3 E**	**DENV-2 E**

E17*^c^*	EDI/II	3.45	3.47
4G2*^d^*	Fusion loop	0.83	0.20
12C1*^d^*	EDIII, not LR	3.72	3.86
E42*^c^*	EDIII	3.09	2.40
E77*^Com^*	EDIII, AS, LR	2.41	1.21
h-23.13*^d^*	EDI/II	0.52	0.10
h-1N5*^d^*	Fusion loop	1.47	0.45
h-1M7*^d^*	Fusion loop	2.48	3.03
h-2J20	EDIII	3.58	3.46

*^a^Determined by indirect ELISA using *P. pastoris*-produced purified DENV E VLPs as coating antigens.*

*^b^The type-specific mAbs used are described in literature: DENV-1 mAbs E24, E29, E37, and E103 (Shrestha et al., 2010); DENV-2 mAb 3H5 (Henchal et al., 1982); DENV-2 mAbs 70, 104, and 106 (Sukupolvi-Petty et al., 2010); DENV-3 mAbs E1, E3, E4, and E12 (Brien et al., 2010); DENV-3 mAb 8A1 (Wahala et al., 2010); DENV-4 mAbs E2, E29, E42, E43, and E88 (Sukupolvi-Petty et al., 2013). Cross-reactive mAbs: E42 (Shrestha et al., 2010), 4G2 (Henchal et al., 1982); E17 and E77 (Brien et al., 2010); 12C1 (Wahala et al., 2010); h23.13 (De Alwis et al., 2011); h-2J20 (Smith et al., 2012); h-IN5 and h-1M7 (Smith et al., 2013). All mAb are murine, except the last four which are human (prefixed ‘h’).*

*^c^Sub-complex-specific mAbs; E17 recognizes EDI/II of DENV-1 and DENV-3; E42 binds EDIII of DENV-1, DENV-2 (weak) and DENV-3.*

*^d^Complex-specific mAbs; 4G2, h-1N5, and h-1M7 bind the fusion loop of all four DENV Es; 12C1 binds recombinant EDIII (outside the LR epitope) of all four DENV serotypes.*

The DENV-3 E VLPs were also recognized efficiently by DENV-3 mAbs E1 (Brien et al., 2010) and 8A1 (Wahala et al., 2010). Both these are type-specific mAbs that manifest binding to EDIII of DENV-3. EDIII contains the lateral ridge (LR) epitope (constituted by *aa* residues 301, 302, 329, 330, and 386) and elicits potent virus-neutralizing antibodies (Gromowski and Barrett, 2007; Shrestha et al., 2010). While mAb E1 binds to an *aa* residue in the vicinity of the LR epitope (*aa* 340), mAb 8A1 interacts with several LR epitope *aa* residues. This is a DENV-3 specific mAb which is reported to neutralize several, but not all, DENV-3 genotypes (Wahala et al., 2010). Consistent with this, mAbs E77 (Brien et al., 2010), 12C1 (Wahala et al., 2010), DV1 E42 (Shrestha et al., 2010), and h-2J20 (Smith et al., 2012) also manifested strong reactivity toward the DENV-3 E VLPs. These mAbs manifest cross-reactivity toward conserved EDIII epitopes. mAb E77 binds to the LR epitope (Brien et al., 2010), while mAb 12C1 binds to EDIII determinants outside the LR epitope (Wahala et al., 2010). Collectively, these data lead to the conclusion that DENV-3 E VLPs largely preserve the antigenic integrity of EDIII. Overall, it can be said that the DENV-3 E VLPs were recognized quite efficiently by mAbs specific to EDI/II, the fusion loop and EDIII.

Finally, the DENV-3 E VLPs were not recognized by any of the DENV-1, DENV-2, or DENV-4 type-specific anti-E or anti-EDIII mAbs specific to serotype 1, 2, and 4 (**Table 1**). That this lack of reactivity was not due to non-functional mAbs was ruled out by testing them against their cognate type-specific antigens. For example, ELISA optical density values for mAbs E24, E29, and E103 with MBP-EDIII-1 as the coating antigen were 3.11, 3.11, and 3.5, respectively. Similarly, mAbs E88 and E423 manifested comparable reactivity toward MBP-EDIII-4 antigen. Overall, the data strongly support the conclusion that the DENV3 E VLPs possess E epitopes essentially typical of DENV-3 serotype.

### DENV-3 E VLPs are Immunogenic and Elicit Virus-Specific Antibodies

**FIGURE 2:**
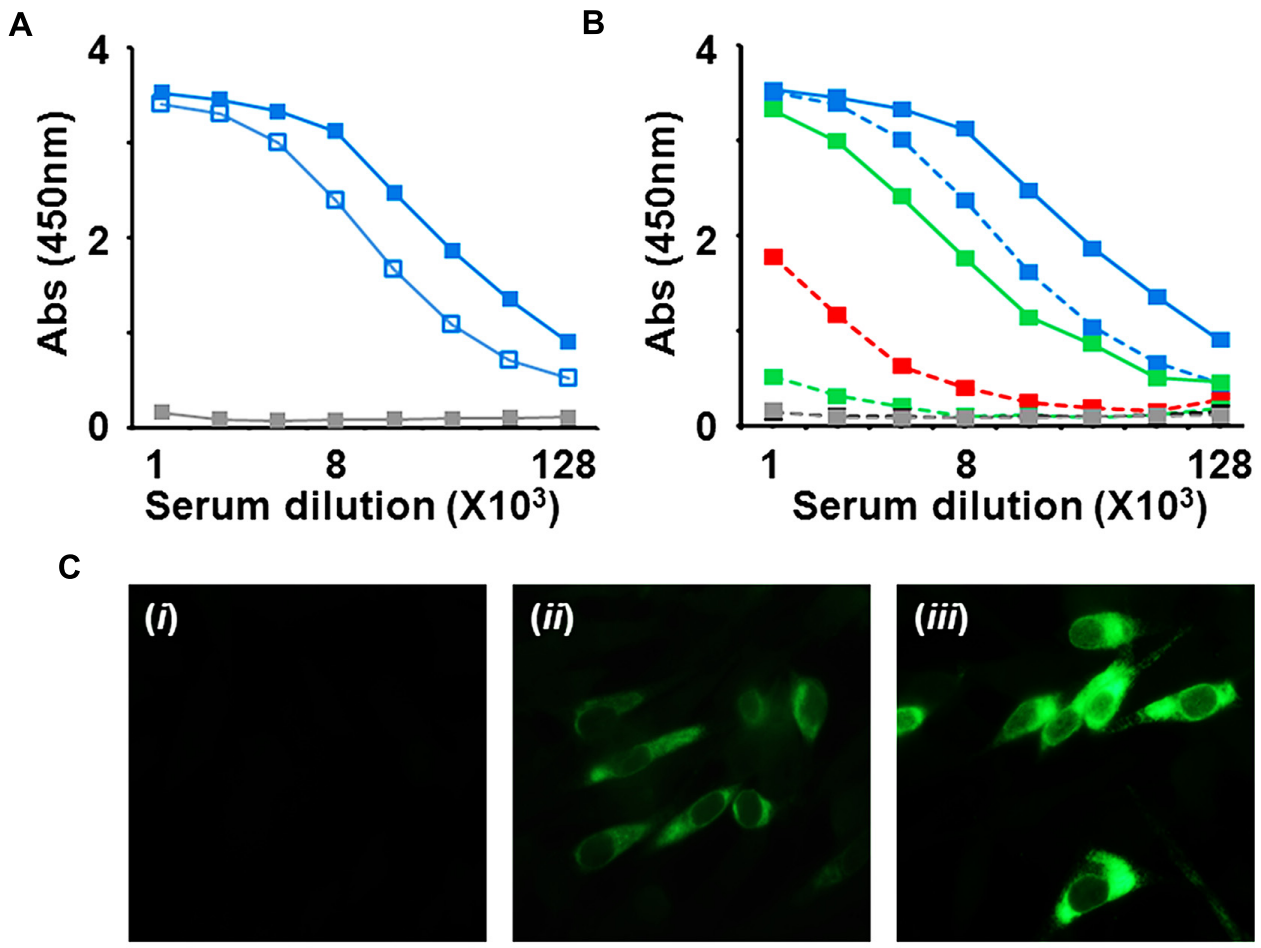
**Preliminary analysis of anti-DENV-3 E VLP induced antibodies. (A)** Pooled sera from DENV-3 E VLP-immunized mice after the first (open blue squares) and second (filled blue squares) boosts and mock-immunized (gray filled squares) BALB/c mice were tested in an indirect ELISA using DENV-3 E protein as the coating antigen. **(B)** Anti-DENV-3 E antiserum obtained after the second boost was tested in ELISAs using DENV-2 E VLPs (solid green), DENV-3 E VLPs (solid blue), recombinant monovalent EDIII-1 (dashed, red), EDIII-2 (dashed, green), EDIII-3 (dashed, blue) or EDIII-4 (dashed, black) proteins as the coating antigens. The ELISA profile of sera from mock-immunized mice (dashed, gray) is found to be superimposed over that of the immune serum against EDIII-4 as the coating antigen. **(C)** Indirect immunofluorescence analysis of DENV-3 virus-infected BHK-21 cells using (i) mock-immunized serum, (ii) 4G2 mAb, or (iii) anti-DENV-3 E antiserum, as the source of primary antibodies. Bound antibodies were visualized using anti-mouse IgG-FITC conjugate.

The immunogenicity of the DENV-3 E VLPs above was evaluated in BALB/c mice using a three-dose regimen as described (Mani et al., 2013). Antibody titers in immune sera collected from these mice were determined using an indirect ELISA, with purified DENV-3 E VLPs as the coating antigen (**Figure 2**). Consistent with that seen earlier for DENV-2 E VLPs, we found an immune boosting effect with DENV-3 E VLPs as well (**Figure 2A**). As a result, the next ELISA was done using sera collected after the second boost. In this experiment, shown in **Figure 2B**, we examined the capacity of DENV-3 E VLP immune sera to cross-react with DENV-2 E VLPs as the coating antigen. This showed that ELISA reactivity toward DENV-2 E VLPs was ~20–25% that seen toward DENV-3 E VLPs. This decrease was statistically significant (*p* < 0.05). Interestingly, ELISA titers observed when the coating antigen used was the recombinant EDIII protein, revealed that antibodies elicited by DENV-3 E VLPs predominantly recognize EDIII-3 as the coating antigen. The reactivity toward EDIII of serotype 2 was very significantly lower (*p* < 0.01), and negligible in the case of EDIIIs of serotypes 1 and 4. This suggests that DENV-3 E VLPs tend to induce largely serotype 3-specific antibodies. As a next step, we investigated if these anti-DENV-3 E antibodies would also recognize and bind to infectious DENV-3, using an indirect IFA. The data in **Figure 2C** show that the DENV-3 E-induced antibodies can efficiently bind to DENV-3 E in infected BHK-21 cells. As in the case of DENV-2 seen earlier (Mani et al., 2013), the immunofluorescence pattern observed using anti-DENV-3 E VLPs antiserum showed DENV-3 replication to be essentially localized to the cytoplasm.

### DENV-3 E VLPs Induce Predominantly EDIII-Specific Homotypic and Potently Neutralizing Antibodies

**FIGURE 3:**
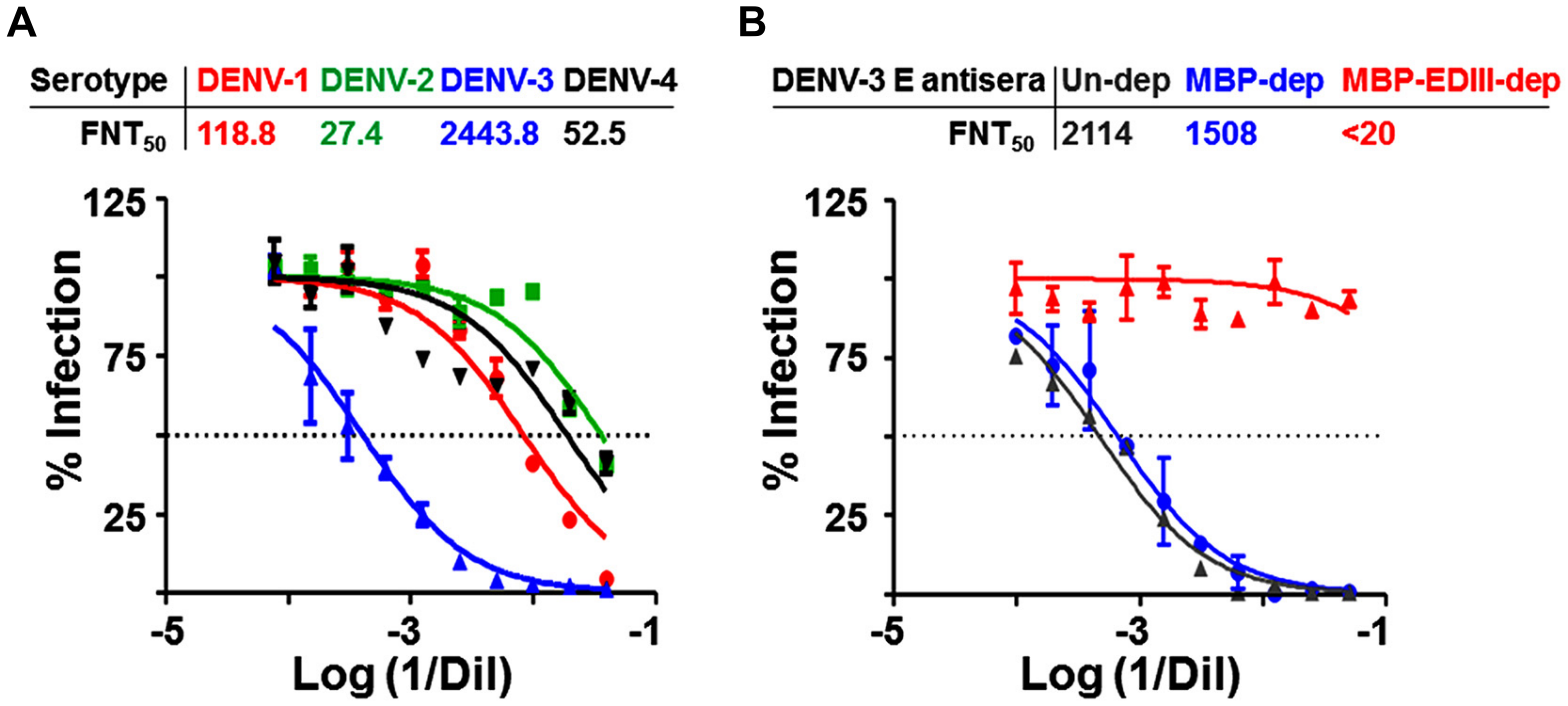
**Characterization of neutralization potency of DENV-3 E VLP induced antibodies. (A)** Two-fold serial dilutions of heat inactivated immune serum from DENV-3 E VLP-immunized mice was assessed for its potency to neutralize and inhibit the infectivity of DENV-1 (red), DENV-2 (green), DENV-3 (blue) and DENV-4 (black) using FACS-based neutralization assay. The y-axis corresponds to the observed percentage of virus infection in Vero cells. The dotted horizontal line represents 50% infection. The *x*-axis corresponds to logarithm of reciprocal serum dilution. **(B)** Effect of EDIII-specific antibody depletion on the DENV-3 virus neutralization potency of anti-DENV-3 E VLP antiserum. The same experiment as shown in panel A, except that the immune serum was pre-depleted with MBP alone (blue) or MBP-EDIII-3 fusion protein (red) before being assessed for its neutralization potency against DENV-3 infection. Control serum (gray) was analyzed in parallel without any pre-depletion with either MBP or MBP-EDIII-3 fusion protein.

Following this preliminary analysis of the antibodies elicited by the DENV-3 E VLPs, we next sought to determine if these could prevent DENV from infecting susceptible cells. To this end, we employed a FACS-based virus neutralization assay, the results of which are depicted in **Figure 3A**. In this experiment we tested the neutralization capacity of the DENV-3 E VLP immune sera against each one of the four WHO reference DENV strains (Kraus et al., 2007). Our data revealed that the anti-DENV-3 E VLP antibodies neutralized only DENV-3 most effectively (FNT_50_ titers > 2400). The remaining three serotypes were only weakly neutralized, with neutralization titers against DENV-1, DENV-2, and DENV-4 being 1–5% the titers observed for DENV-3. Heterotypic neutralization titers were very significantly lower compared to homotypic neutralization titers (*p* < 0.0001).

Given that the previous ELISA data (**Figure 2B**) showed the predominant reactivity of DENV-3 E VLP-induced immune sera to EDIII-3, we sought to understand the role of these antibodies in the DENV-3 virus-neutralizing activity observed above (**Figure 3A**). To this end, we performed an antibody depletion experiment, as shown in **Figure 3B**. In this experiment, we selectively depleted the EDIII-specific antibodies in the immune serum by pre-incubating it with recombinant EDIII-3 protein (as an MBP fusion) and then determined the residual DENV-3 neutralizing antibody titer. While a control depletion (performed with MBP alone) produced a marginal decrease in virus neutralizing antibody titer, depletion with EDIII-3 resulted in a complete abrogation of virus neutralizing activity. Our results very strongly support the conclusion that the DENV-3 neutralizing antibodies present in the DENV-3 E VLP-induced immune serum are exclusively directed toward EDIII.

**FIGURE 4:**
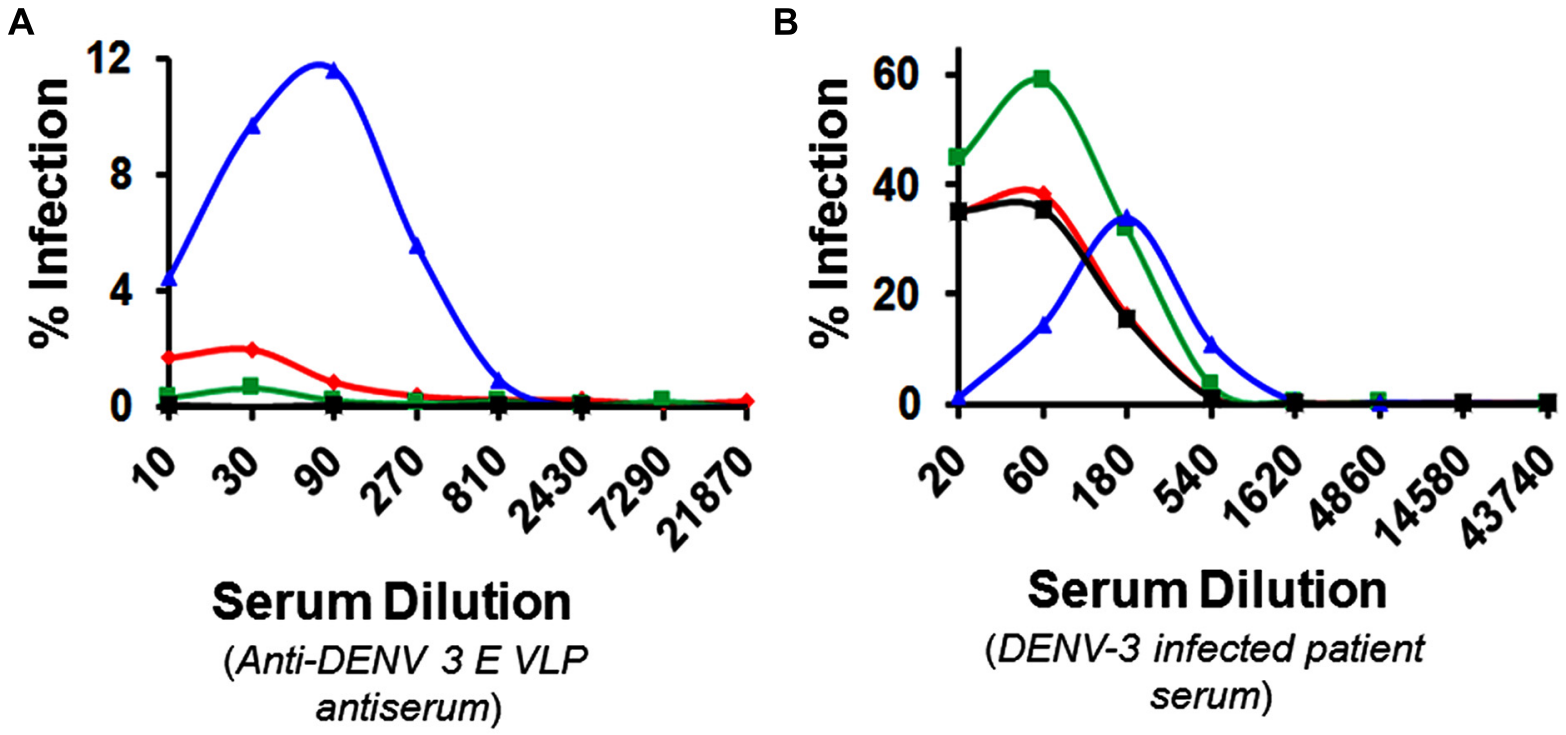
**Antibody-dependent enhancement analysis.** Serial dilutions (*x*-axis) of heat-inactivated anti-DENV-3 E VLP immune serum **(A)** or patient serum (primary DENV-3 infection) were analyzed for their ability to promote uptake (*y*-axis) of DENV-1 (red), DENV-2 (green), DENV-3 (blue) or DENV-4 (black) into Fcγ-bearing K562 cells, using a FACS-based enhancement assay. Note that *y*-axis in **(B)** is at a fivefold bigger scale compared to that in **(A)**. Data from one of two independent experiments are shown.

That the *P. pastoris*-produced DENV-3 E VLPs contain epitopes recognized by cross-reactive mAbs (**Table 1**) and the antiserum raised using these VLPs contains cross-reactive antibodies (**Figure 2B**) is evident from our observations above. Would these cross-reactive antibodies play a role in facilitating uptake of heterotypic DENVs through the Fc receptor pathway? To address this, we examined the potential of the DENV-3 E VLP-induced antibodies to cause enhancement. Fcγ receptor-bearing K562 cells were infected with DENVs in the presence of varying dilutions of the anti-DENV-3 E VLP antiserum, followed by determination of the proportion of cells infected as a function of immune serum dilution. For a comparison, serum from a DENV-3 infected patient was also used in a parallel experiment. From the results (**Figure 4**) it is evident that the patient serum manifested significant heterotypic enhancement while the DENV-3 E VLP-induced immune serum did not. A 20-fold dilution of the patient serum resulted in ≥35% K-562 cells becoming infected with DENV-1, -2, and -4 (heterotypic enhancement) with virtually no DENV-3 infection. In contrast, a 20-fold dilution of anti-DENV-3 E VLP antiserum manifested virtually insignificant heterotypic enhancement effect on DENV-1, -2, and -4, and a low level of homotypic enhancement of DENV-3 infection.

## Discussion

We recently showed that a carboxy-terminally truncated version of the E protein of DENV-2 expressed in *P. pastoris* could assemble into discrete VLPs in the absence of any prM expression. That these VLPs could elicit predominantly DENV-2 virus-specific homotypic neutralizing antibodies underlined their potential as alternate monovalent DENV-2 vaccine candidate (Mani et al., 2013). Implicit in this finding is the possibility that one could envisage a tetravalent DENV vaccine formulation containing DENV E ectodomain-based VLPs of all four serotypes. Realizing this possibility is contingent upon the following three pre-requisites: (i) the E ectodomains of other DENV serotypes expressed in *P. pastoris* should possess similar VLP-forming potential; (ii) these VLPs should elicit high titer serotype-specific neutralizing antibodies; (iii) further, these VLPs should not manifest any potential for ADE. To this end, we investigated the antigenic properties and immunogenicity of the E ectodomain of a second DENV serotype, DENV-3, in this paper.

We expressed DENV-3 E ectodomain (first 393 *aa* residues), flanked by DENV-3 prM-derived signal peptide at the N-terminus and a 6× His tag at the C-terminus, in *P. pastoris* and purified it as before under denaturing conditions using Ni^2+^-NTA affinity chromatography. The signal peptide was found to be appropriately cleaved off and the protein was found to be glycosylated as observed earlier for DENV-2 E (Mani et al., 2013). Importantly, and as anticipated, the purified DENV-3 E ectodomain protein was assembled into VLPs based on EM and DLS analyses. This, taken together with our previous work with DENV-2 E (Mani et al., 2013) and studies on HBsAg (Lunsdorf et al., 2011), strongly suggest that that VLP assembly of *P. pastoris*-produced viral antigens occurs post expression during downstream processing.

Do the E VLPs maintain the antigenic integrity of E and its domains especially as prM is absent? This was addressed by epitope mapping using a battery of type-specific and cross-reactive human and murine mAbs. Epitopes on EDI/II were recognized by several serotype-specific mAbs (DENV-3 E mAbs E3, E4, E12) as well as cross-reactive mAb E17. Reactivity of mAbs 4G2, mAb h-1N5 and mAb h-1M7, indicated that the fusion loop is intact. Importantly, epitopes of EDIII implicated in the induction of type-specific neutralizing antibodies were displayed appropriately on the surface of the DENV-3 E VLPs based on the reactivity toward DENV-3 E mAb 8A1 (Wahala et al., 2010), E77 (Brien et al., 2010), 12C1 (Wahala et al., 2010), and DV1 E42 (Shrestha et al., 2010). All these antibodies recognize epitopes in EDIII. The LR epitope, the major EDIII target of strong neutralizing antibodies, is apparently intact based on the reactivity of mAbs such as E77 and 8A1. The antigenic structure of EDIII outside the LR is also preserved as the DENV-3 E VLPs react with mAb 12C1, whose binding to EDIII is unaffected by point mutations in LR epitope (Wahala et al., 2010). The antigenic structure analysis is consistent with the *P. pastoris*-produced DENV-3 E VLPs retaining the antigenic integrity of the viral E antigen, particularly the EDIII, which is critical in the induction of virus-neutralizing antibodies.

If the DENV-3 E VLPs indeed preserve antigenic integrity of the E antigen and its sub-domains, especially EDIII, would they elicit DENV-3 virus-specific antibodies? Would these be capable of neutralizing DENV-3 infectivity? Our data show that the *P. pastoris*-produced DENV-3 E VLPs are highly immunogenic and elicit antibodies that specifically recognize and bind to DENV-3 in infected cells. Further, in a FACS-based neutralization assay, these antibodies manifested potent DENV-3 virus-neutralizing activity. The remarkable finding was that the antiserum neither manifests significant levels of cross-reactivity in ELISAs nor cross-neutralization of heterologous DENV serotypes in the FACS assay. Significantly, selective depletion of EDIII-specific antibodies from the immune serum resulted in loss of DENV-3 neutralizing potency. Apparently, the neutralizing activity elicited by DENV-3 E VLPs is solely associated with the EDIII-specific fraction of the polyclonal response. This conclusion is consistent with the notion that the DENV-3 E VLPs presumably display EDIII efficiently on the VLP surface. That DENV-2 E VLPs also elicit predominantly homotypic neutralizing antibodies (Mani et al., 2013), is consistent with this notion.

What is the infection-enhancing potential of the anti-DENV-3 E VLP antibodies? As these VLPs do not contain prM, we may rule out the elicitation of anti-prM antibodies implicated in ADE (Dejnirattisai et al., 2010; Rodenhuis-Zybert et al., 2010) of DENV infection. However, the identification of several cross-reactive epitopes on the DENV-3 E VLPs (**Table 1**) suggests that these can elicit cross-reactive antibodies. This is consistent with the observed cross-reactivity of anti-DENV-3 E VLP antiserum in ELISAs using DENV-2 E VLPs and heterologous recombinant EDIIIs as coating antigens. Significantly, an ADE assay using K562 cells, demonstrated that the anti-DENV-3 E VLP antiserum did not possess discernible ADE activity on the infectivity of heterologous DENV serotypes. This is consistent with the potent and type-specific neutralizing antibody response elicited by the DENV-3 E VLPs. We believe the observed low levels of homotypic ADE to be an experimental phenomenon which may be physiologically irrelevant. This notion draws support from recent work using an *in vivo* ADE model system which shows that type-specific neutralizing antibodies do not enhance infection at any concentration (Watanabe et al., 2015).

This work extends and strengthens our previous observation that the DENV E ectodomain expressed in *P. pastoris* can assemble into immunogenic VLPs. These VLPs largely retain the antigenic architecture of the viral epitopes critical for the induction of potent virus-neutralizing antibodies. An analysis of the epitope architecture on the VLP suggests that antigenic integrity of the E protein is intact but subtly altered in way that presents EDIII in a more accessible manner on the VLP surface. The *P. pastoris*-produced DENV-3 VLPs presumably serve as an efficient EDIII-display platform. This we believe underlies the predominantly homotypic and EDIII-specific neutralizing antibody response with inherently low potential for ADE.

## Conclusion

The demonstration that DENV VLPs with promise of efficacy and safety can be produced using *P. pastoris*, an expression system that offers several key advantages from the perspective of inexpensive vaccine production, in resource-poor countries where dengue is endemic, strongly warrants further exploration of this approach.

## Author Contributions

LT and SM: performed cloning, expression and purification work; RR: performed ELISA, FNT, antibody depletion and ADE assays; AP: helped with FNT and ADE assays; PT: carried out the EM and DLS characterization work; UA: Co-ordinated immunization work and performed IFA. AdS helped design FACS-based experiments and helped with data analysis. SS and NK: Conceived and designed the work, analyzed the data and wrote the final manuscript. All authors provided inputs for the initial draft and approved the final version.

## Conflict of Interest Statement

The authors declare that the research was conducted in the absence of any commercial or financial relationships that could be construed as a potential conflict of interest.
