# Supplementary material for: Pichia pastoris-expressed dengue 3 envelope-based virus-like particles elicit predominantly domain III-focused high titer neutralizing antibodies
Source: Front Microbiol. 2015 Sep 23;6:1005. doi: 10.3389/fmicb.2015.01005 (PMC4585145; doi:10.3389/fmicb.2015.01005)
Supplement: Supplementary file 1 [file Image_1.PDF]

Supplementary Data

Pichia pastoris-expressed Dengue 3 Envelope-based Virus-like Particles Elicit Predominantly Domain III-Focused High Titer Neutralizing Antibodies

Lav Tripathi, Shailendra Mani, Rajendra Raut, Ankur Poddar, Poornima Tyagi, Upasana Arora, Aravinda de Silva, Sathyamangalam Swaminathan and Navin Khanna

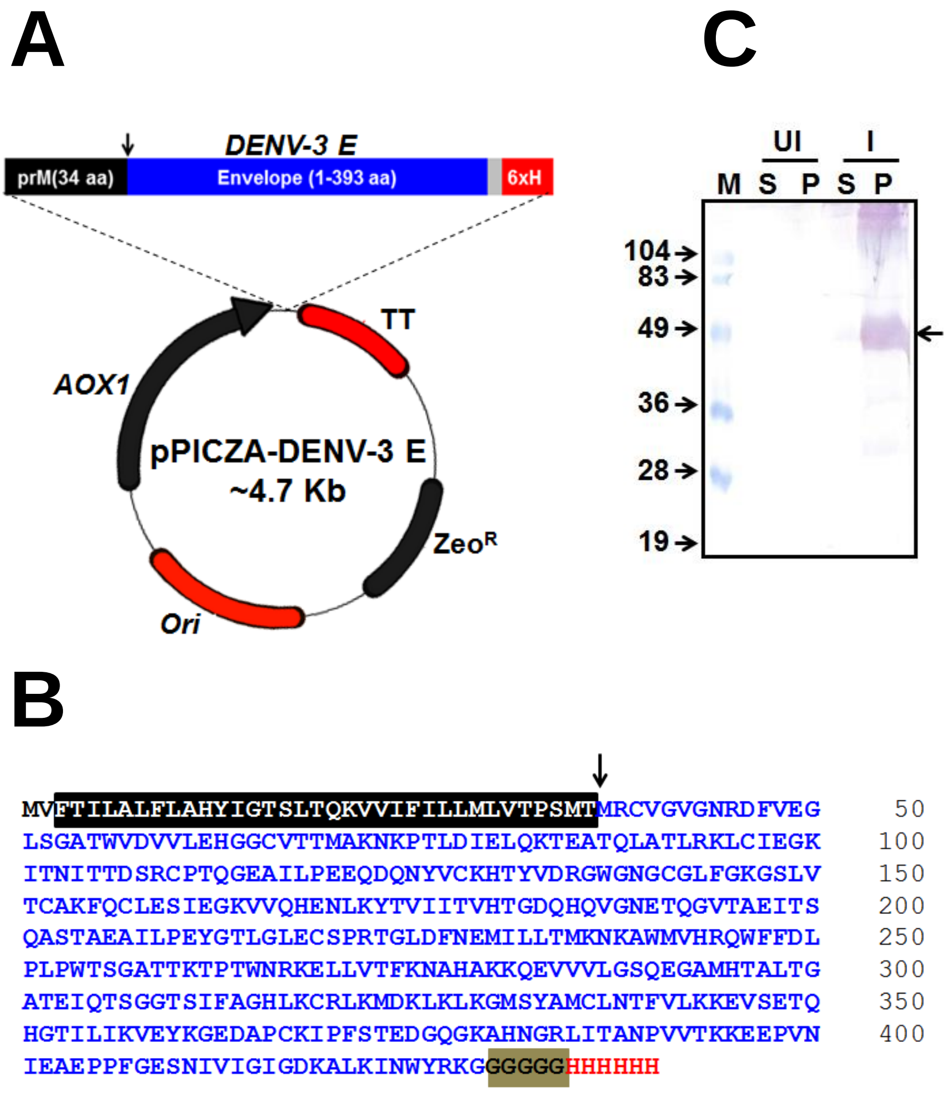

Figure S1: Design and expression of recombinant DENV-3 E antigen. (A) Map of the DENV-3 E expression plasmid integrated into *P. pastoris* genome. The *DENV-3 E* gene was cloned between the *AOX1* promoter and the *AOX1* transcriptional terminator (TT). Zeo^R and *Ori* denote the zeocin selection marker and the plasmid origin of replication, respectively. The inserted gene encodes the first 393 *aa* residue DENV-3 E (ectodomain), preceded by the C-terminal 34 *aa* residues of DENV-3 prM. The grey box denotes the pentaglycine linker

peptide joining the C-terminus of E ectodomain to the polyhistidine tag (6×H). (B) The predicted *aa* sequence of the DENV-3 E antigen. Kozak consensus sequence-encoding nucleotides resulted in the addition of two extra *aa* residues (MV) in front of the prM signal peptide. The color scheme corresponds to that shown for the *DENV-3 E* gene insert in ‘A’. The downward arrows in ‘A’ and ‘B’ denote the signal cleavage site. (C) Western blot analysis to localize DENV-3 E antigen expression in induced *P. pastoris*. Proteins in lysates of un-induced (UI) and induced (I) *P. pastoris* cultures were separated into soluble (S) and membrane-enriched pellet (P) fractions, followed by SDS-PAGE and immunoblot analysis using mAb 24A12. Pre-stained protein markers were analyzed in lane ‘M’. Their sizes (in kDa) are indicated to the left. The arrow on the right indicates the position of the recombinant DENV-3 E antigen.

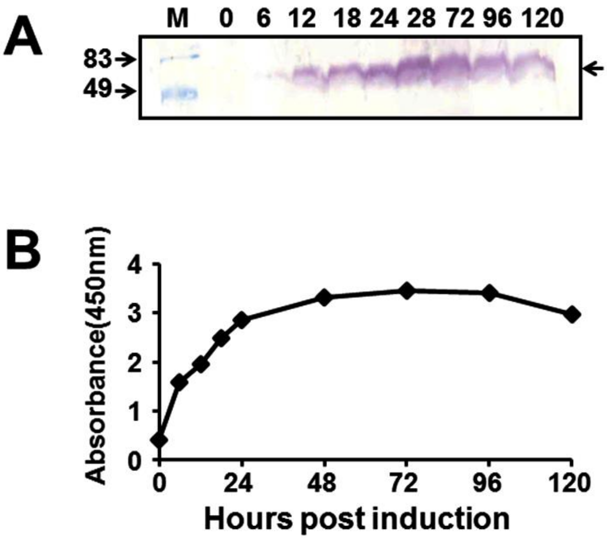

Figure S2: Optimization of induction time for DENV-3 E expression. (A) Detection of DENV-3 protein in methanol-induced *P. pastoris* cultures. Induction was carried out using 1% methanol for varying time durations (0-120h). Equivalent aliquots of induced cultures (normalized based on culture OD_{600}) were lysed and centrifuged to obtain the membrane-enriched ‘P’ fractions. These were solubilized in 8M urea-MEB, separated on SDS-polyacrylamide, electro-transferred onto nitrocellulose and probed with mAb 24A12. DENV-3 E protein was visualized using anti-mouse IgG HRPO/TMB substrate. (B) Ni^{2+}-NTA His-Sorb ELISA. Urea solubilized ‘P’ fractions from A were bound to microtiter wells of His-Sorb plates. After washing away unbound material, bound DENV-3 E was detected using the same set of primary and secondary antibodies as in Western except that the developed colored product was solubilized in sulphuric acid, followed by measurement of absorbance at 450nm.

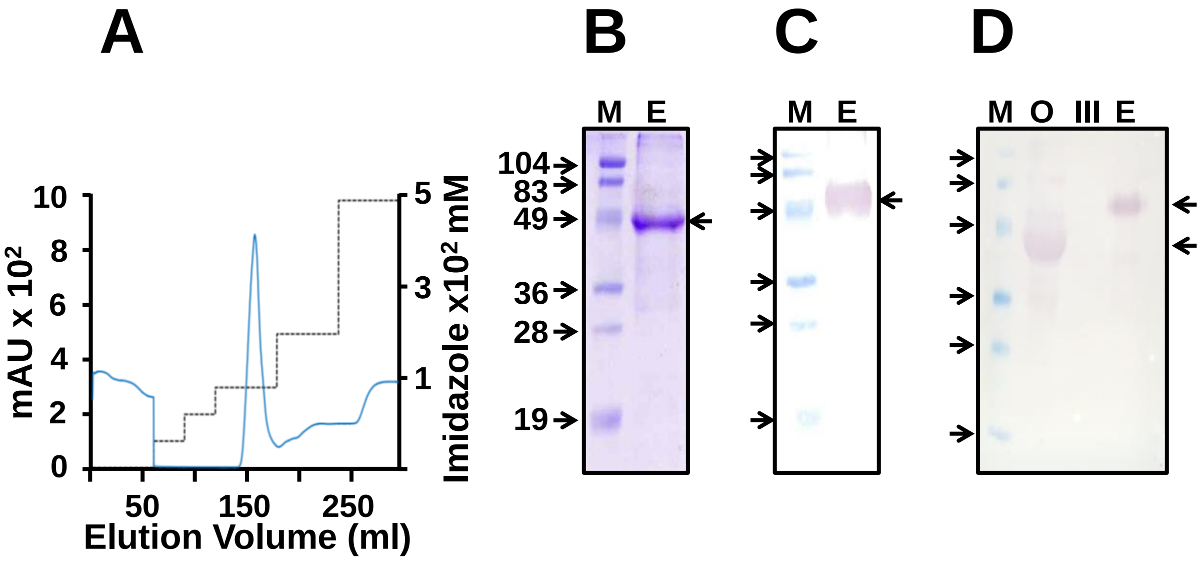

Figure S3: Purification and characterization of recombinant DENV-3 E antigen. (A) Ni^{2+} affinity chromatographic purification of DENV-3 E antigen from the 'P' fraction of induced *P. pastoris* lysate. The solid and dashed curves represent the profiles of UV absorbance (at 280 nm) and the imidazole step gradient, respectively, during chromatography. (B) Coomassie-stained SDS-polyacrylamide gel analysis of the pooled purified protein (Lane 'E'). (C) Immunoblot analysis of the purified protein using mAb 24A12. (D) Protein blot using Con A-HRPO conjugate. Lane 'E' denotes the purified DENV-3 E protein (pooled peak material shown in panel 'A'). In lanes 'O' and 'III' ovalbumin and purified EDIII-3 protein (lane 'III'), were analysed as positive and negative controls, respectively. In panels, B-D, protein markers (whose sizes, in kDa, are shown to the left of each panel) were run in lanes 'M'. The arrow to the right of each panel indicates the position of the recombinant DENV-3 E antigen.

***
